# Position and Direction Tracking of a Magnetic Object Based on an M_x_-Atomic Magnetometer

**DOI:** 10.1038/s41598-020-57923-w

**Published:** 2020-01-28

**Authors:** Asieh Soheilian, Maliheh Ranjbaran, Mohammad Mehdi Tehranchi

**Affiliations:** 1 Laser and Plasma Research Institute, Shahid Beheshti University, Tehran, Iran; 2 Physics Department, Shahid Beheshti University, Tehran, Iran

**Keywords:** Physiology, Materials for optics

## Abstract

Remote and non-invasive tracking of a moving magnetic object based on an atomic magnetometer has been developed recently. The sensitivity of atomic magnetometers is limited by mechanisms that relax the spin precession of alkali atoms. Meanwhile, some of these mechanisms such as magnetic field gradient are applicable in magnetic object tracking. Correspondingly, we have illustrated a way of operating an M_x_ atomic magnetometer to measure the magnetic field and its gradient simultaneously for a moving magnetic microwire, which resulted in recording a spike-like signal. We described the dependency of the signal on the position, velocity, and direction of the microwire. According to the results, the measurement of the inhomogeneous local magnetic field gradient opens new ways for obtaining the direction of the velocity of magnetic objects accessible in cells with large sizes. Furthermore, the accuracy of the velocimetry was found as 40 µm/s which could be an important means for assessing the microvascular blood flow.

## Introduction

Precise tracking of a magnetic object has various applications ranging from industrial^1^ to biomedical applications^2^. The parameters of this tracking, such as position, direction, orientation, and velocity have been obtained based on measuring the object’s magnetic field and its spatial gradients. The magnetic field’s accurate mapping has been the base of the most recent magnetic trackers which could be measured by various magnetic sensors^3^. Most of these sensors developed so far could detect the magnetic field and its spatial gradients using conventional induction coils^4,5^. The main drawback of these induction sensors was that their frequency must be large enough to satisfy signal-to-noise considerations. From a practical point of view, in biomagnetism, the frequency increment has two health effects on the human body. One of them is called tissue heating (or specific absorption rate (SAR)) and the other one is peripheral nerve stimulation (PNS)^6^. In order to reduce these health effects, a low-frequency magnetic sensor should be used such as giant magneto resistive sensors (GMRs)^7,8^, integrated giant magnetic impedance sensors (GMIs)^9^, tunneling magneto resistive sensors (TMRs)^10^, fluxgates^11^, superconducting quantum interference devices (SQUIDs)^12,13^, and atomic magnetometers^14^. The main drawback of the GMR, GMI, TMR, magnetoelectric sensor, and fluxgates is that due to their low sensitivity, they need to be close to the magnetic objects. SQUIDs have been established for the past few decades as the most sensitive magnetic field detectors. Meanwhile, atomic magnetometers have attracted substantial interest over the past years which compete with SQUID detectors because of their high sensitivity below f  T/√Hz and no cryogenic requirements for operation. Table 1 compares different available magnetometers in magnetic tracking. These magnetometers have been designed for detection of nano and micro magnetic samples.

**Table 1:** Comparison of different available magnetometers in magnetic tracking.

Sensor	Sensitivity	The accuracy of the measurement	Magnetic sample	Information	Measured parameters	Ref.
Received coil	—	7 mm maximum error	RFID tag*	Localization of a magnetic dipole with a simple algorithm	Magnetic field, nondiagonal, and diagonal components of the gradient tensor	^3^
Fluxgate	10^-12^ T	0.2–2.7% maximum error	Magnetite powder	3D MPG**	Remnant magnetic fields	^11^
Atomic magnetometer	16.9 fT/ √Hz along the y-axis and 16.7 fT/√ Hz along the z-axis	Sub-picomolar	30 nm ferromagnetic iron and cobalt nanoparticles	Real-time surveillance of the magnetic separation of nanoparticles from water and whole blood	Magnetic field	^2^
GMI	1 nT/*√*hz	Concentration as small as 5.47 × 1^–9^ mol	20 nm iron oxide	Analysis of blood samples	Magnetic field	^9^
GMR	50 nT/*√*hz	10^4^ cells/ml	Magnetically labeled individual cells	Flow cytometry	Magnetic field	^8^
SQUID	3 fT/ √hz	Millimeter/millisecond	Single microsphere	Evaluation diameter constrictions in an arteria phantom	Magnetic field	^13^

^*^Radio frequency identification (RFID) tag.

^**^A non‐invasive method for pneumoconiosis diagnosis.

Some past research employed an atomic magnetometer for tracking magnetic particles. A first-order gradiometer with sensitivity of 100 fT/√Hz was used to detect a 150 µm diameter cobalt microparticle^15^. Maser *et al*. improved the sensitivity of the atomic magnetometer approximately by 2 orders of magnitude through working in the spin-exchange relaxation free (SERF) regime which resulted in tracking of a single ferromagnetic cobalt particle with a diameter of 2 µm^16^. Recently, researchers have shown improved sensitivity for tracking ferromagnetic iron and cobalt nanoparticles which flowed in water and blood^2^. There has also been some research to apply atomic magnetometer for magnetic particle imaging (MPI) techniques^17^. Colombo *et al*. estimated that the atomic magnetometer could detect iron down to 1 *µ*g by their MPI^18^.

The atomic magnetometer’s sensitivity is limited by any mechanism which relaxes the spin of alkali atoms. The relaxation mechanisms reduce the expectation value of the spin component along the direction of the magnetic field and dephase the precessing atoms, in longitudinal and transverse relaxation times, respectively^19–22^. In general, one of the dephasing contributions to the transverse relaxation rate is the magnetic field inhomogeneities^23–28^. Such inhomogeneities cause the loss of phase coherence in atoms as these atoms experience the changes in the magnetic field when they are diffused throughout the cell. Note that eliminating the magnetic field inhomogeneities can improve the sensitivity, and its measurement can be important in some applications such as magnetic resonance imaging (MRI)^29^, nuclear magnetic resonance (NMR) experiments^30^, and magnetic object tracking^3^.

Thus, the transverse relaxation rate can be used as an observable variable to measure the changes in the inhomogeneous local magnetic field gradient. In previous papers, several sensors such as SQUIDs gradiometers^31,32^, atomic magnetometers^33^, or NMR techniques^34^ have been used to readout directly the spin precession signal. The signal provides direct access to the transverse relaxation rate (or magnetic field inhomogeneities) by measuring the amplitude of the exponential decay. Nevertheless, in this paper, we extracted the relaxation rate changes by observing the widths of the resonance signals. This kind of measurement was fully executable and analyzable in cells with a large size. Accordingly, the simultaneous changes of the magnetic field and inhomogeneous local gradient field generated by a moving magnetic object were detected as a spike-like signal. The aim of this study was to extract the tracking information from this received signal. We have studied the dependency of the output signal on the velocity and size of the moving microwire. We incorporated the experimental analysis with finite element method simulation. Experimental results were qualitatively in good agreement with simulation results. Finally, we have also demonstrated the advantages and limitations of utilizing the large alkali vapor cell size in the magnetic tracking.

## Materials and Methods

### Experimental setup

**Figure 1:**
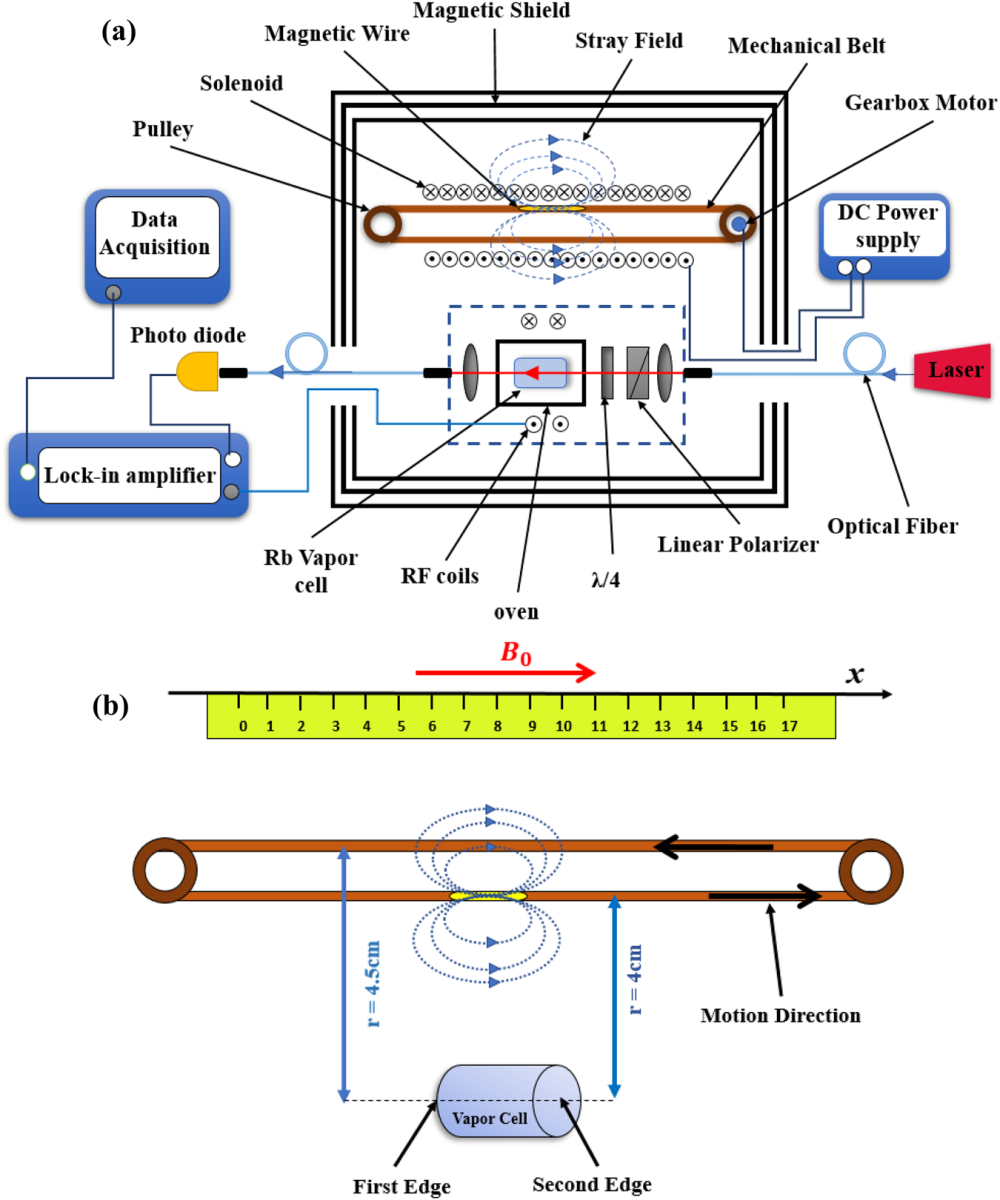
(**a**) The experimental set-up for tracking a moving magnetic microwire consisting of an M_x_-atomic magnetometer and a mechanical transmission line; (**b**) The sketch of the mechanical transfer path and the relative position of the magnetic microwire to the Rb vapor cell; The horizontal position was obtained from the graded path and the vertical position was 4 cm when the microwire moved along the bias magnetic field and 4.5 cm when it moved in the opposite direction of the magnetic field. The location of the first and second edges of the vapor cell were at 5.5 cm and 10.5 cm relative to the origin (x = 0), respectively.

Among the highly sensitive magnetometers, atomic magnetometers are generally presumed as one of the best candidates to detect infinitesimal changes in the magnetic field and its inhomogeneous local gradient generated by a moving magnetic object^15,16^. In order to detect the moving magnetic object, we used an M_x_-atomic magnetometer as sketched in Fig. 1(a)^20,21,35–38^. The atomic magnetometer was mounted inside of a three-layer µ-metal magnetic shielding providing a passive reduction of the magnetic field. This atomic magnetometer relied on the creation of spin polarization in a ^85^Rb atomic vapor cell which was cylindrical in shape, with 2.5 cm diameter and 5 cm height. The vapor cell was coated with Octadecyltrichlorosilane (OTS) and filled with 10 torr N_2_ gas which is crucial in quenching excited atoms. It was electrically heated to around 60 °C. Electron spin polarization was generated by passing a circularly polarized beam through the Rb vapor cell. The laser beam was guided from a distributed feedback (DFB) laser while being actively stabilized to the D_1_ transition 794.8 nm (F = 3 → F′ = 2) by a Doppler-free method. Polarized spin atoms of the medium reached a steady state under the torque exerted by B_0_ as well as by relaxation mechanisms. Under such conditions, precession of spins occurs around B_0_, according to the relation ω_L_ = γB_0_, where is the gyromagnetic ratio and B_0_ denotes the bias magnetic field. To maintain spins in a phase coherent manner, the spin polarization was coherently driven by a weak oscillating magnetic field denoted by B_rf_. This field was produced by a pair of small Helmholtz coils whose current was generated by a built-in oscillator of a lock-in amplifier (Stanford Research Systems, SR830). When the oscillating magnetic field is driven at Larmor frequency, ω_L_, this leads to a magnetic resonance and a modulation in the transmitted light intensity. The modulated light was detected and amplified by a photodetector (2031 New Focus) and fed into the lock-in amplifier to extract the in-phase and quadrature signals. This process was fully controlled by a LabVIEW program (Fig. 1(a))^38^.

Meanwhile, we used a mechanical transfer path to move the magnetic microwire in a single direction consisting a mechanical belt looped over two pulley systems (Fig. 1(a)). The pulley transferred the power of a gearbox motor to guide the belt in x-direction at a constant velocity. For tuning the object velocity, we used a motor with a tunable voltage within the range of 0–20 V. The entire transfer system, consisting of the belt and the pulley, were placed inside a 36 cm long solenoid (with 2 cm diameter and 2500 turns).

To detect a moving object by an atomic magnetometer, we needed a soft magnetic material with a high permeability. For this purpose, we used an amorphous microwire with the chemical composition of Co_68.15_ Fe_4.35_ Si_12.5_ B_15_, 1 cm length and 120 µm diameter. The hysteresis loop of the magnetic microwire had already been obtained in previous investigations^39,40^. The results indicated that its saturation magnetization was about 1.9 mT. To magnetize this microwire, the solenoid created a 4 mT magnetic field. The stray magnetic field of the solenoid on the cell location was considered as a bias magnetic field, B_0_, which was far greater than the magnetic field of the microwire, δB_Wire_. In general, the M_x_ atomic magnetometer is scalar in nature, but it is sensitive only to the projection of δB_Wire_ on B_0_ which makes it as a vector magnetometer^17^. When the microwire moved along the bias magnetic field, the vertical position of the microwire relative to the cell was 4 cm, and when it moved in the opposite direction with respect to the magnetic field, the vertical position was 4.5 cm (as shown in Fig. 1(b)).

### Finite element simulation

A moving magnetic microwire creates a magnetic field and an inhomogeneous local gradient field around itself which changes during its movement. To obtain the magnetic field and gradient field of the microwire, a classical magnetometer can be used in combination with a gradiometer respectively. Nevertheless, to simulate these parameters, we performed a 3D modelling through finite element based COMSOL Multiphysics 5.2.1 software using Magnetic Fields, No Currents mode of the AC/DC module.

**Figure 2:**
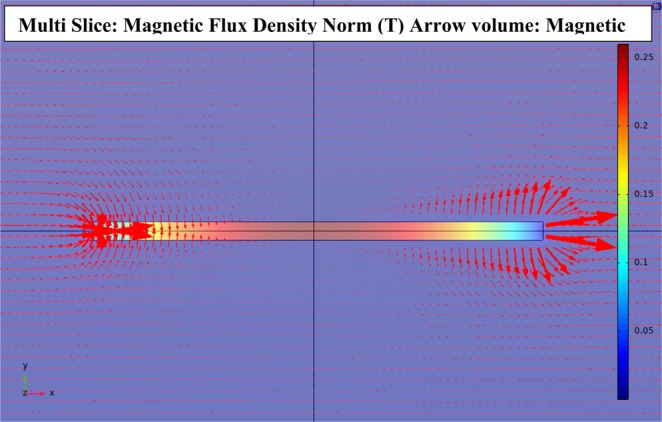
Simulated distribution of the magnetic flux density norm and the magnetic field of the microwire.

Similar to real situations, an x-oriented soft magnetic microwire was placed 4 cm above the vapor cell, and in a 4 mT homogeneous magnetic field in different positions along the x-direction. Figure 2 depicts the simulated distribution of the magnetic field norm around the magnetic microwire.

We calculated the averaged X, Y, and Z magnetic field components for different positions of the microwire. The magnetic field was averaged throughout the length of the cell, L, because in a buffer gas free anti-relaxation coated cell, the alkali atoms perceive an average of the magnetic field throughout the cell. The reason is that the pressure is low and spins diffuse rapidly through the gas. So, the alkali atoms move freely throughout the entire cell^24,25,27^. Based on this fact, we averaged the magnetic field throughout the length of the cell, L.

For further analysis, the inhomogeneous local magnetic field gradient of the microwire was averaged throughout the length of the cell along the X, Y, and Z directions.

## Results and Discussion

### The output signal characteristics of the atomic magnetometer

Before tracking a magnetic object, it is necessary to know the output signal characteristics of the atomic magnetometer. Atomic magnetometers perceive the magnetic field through measuring the Larmor frequency of the electron spin polarization, S = (S_x_, S_y_, S_z_) precessed around the magnetic field axis. In the classical picture, the total time evolution of the polarized spin atoms, S, is described phenomenologically by Bloch equations in the presence of the bias magnetic field B_0_, the oscillating magnetic field B_rf_ (t), as well as the longitudinal (T_1_) and the transverse (T_2_) relaxation times (T_1_ and T_2_ are the rates of the atomic coherence distortion):

1 $$\frac{{\rm{dS}}}{{\rm{dt}}}=-{\rm{\gamma }}{\rm{S}}\times ({{\rm{B}}}_{0}+{{\rm{B}}}_{{\rm{rf}}})-\frac{{{\rm{S}}}_{{\rm{z}}}}{{{\rm{T}}}_{1}}-\frac{{{\rm{S}}}_{{\rm{x}},{\rm{y}}}}{{{\rm{T}}}_{2}}$$

**Figure 3:**
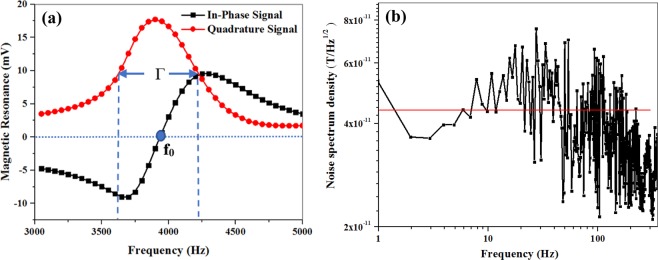
(**a**) In-phase (black squares) and quadrature (red circles) components of the magnetometer resonance signal measured in the laboratory frame; f_0_ is the frequency in the near zero crossing of the in-phase curve which is relevant to the magnetic resonance frequency of the spins. Γ represents the full-width half maximum (FWHM) of the quadrature signal which equals the peak-to-peak frequency interval of the in-phase signal. solid lines are a guide for the eye. (**b**) Noise spectral density of the magnetometer signal.

Where, γ denotes the gyromagnetic ratio of the alkaline metal (Rb: ± 2π × (2.8 MHz/G)/(2I + 1); the sign depends on the hyperfine level F = I ± 1/2). By solving the Bloch equations in a rotating frame of reference, the in-phase S′_y_ and quadrature S′_x_ components of the transverse spin polarization and the longitudinal spin polarization (S_z_) are obtained. Figure 3(a) shows the magnetic resonance line-shapes obtained by altering the frequency of rf magnetic field across the Larmor frequency. In this figure, the in-phase and quadrature output of the lock-in amplifier are in accordance with S′_y_ and S′_x_, the laboratory frame responses of the atomic magnetometer (S_x_ = S′_x_ cos(ωt)+S′_y_ sin(ωt))^38^.

To determine the atomic magnetometer sensitivity, we measured its noise spectral density. It was obtained by recording the atomic magnetometer signal retained on the zero-crossing slope of the in-phase signal as marked by f_0_ in Fig. 3(a). The fast Fourier transform of the magnetic signal created the voltage power spectrum. The voltage power spectrum yielded the magnetic noise spectral density using the frequency calibration of the slope of the in-phase component. The sensitivity of the atomic magnetometer is marked with a solid line in Fig. 3(b).

For utilizing the atomic magnetometer in magnetic tracking applications, a non-distorted output signal is required. So, it is essential to investigate the linearity of the atomic magnetometer response. Our atomic magnetometer had a linear response between its dynamic range. Also, a quantitative measurement of the amplitude of output signal should have been done in response to the input magnetic fields of different frequencies. We extracted this relation for our atomic magnetometer within the range of DC to 50 Hz^37^.

### Detection of the moving magnetic object

Our measurements were performed on a moving magnetic microwire. The microwire was loaded on a 70 cm belt which was driven by a 5 V DC motor. The microwire travelled in the solenoid at a velocity of 1.37 cm/s and was magnetized by a 4 mT magnetic field (as shown in Fig. 1(a)). The stray magnetic field (B_0_) of the solenoid on the cell location was calculated as 820 nT.

**Figure 4:**
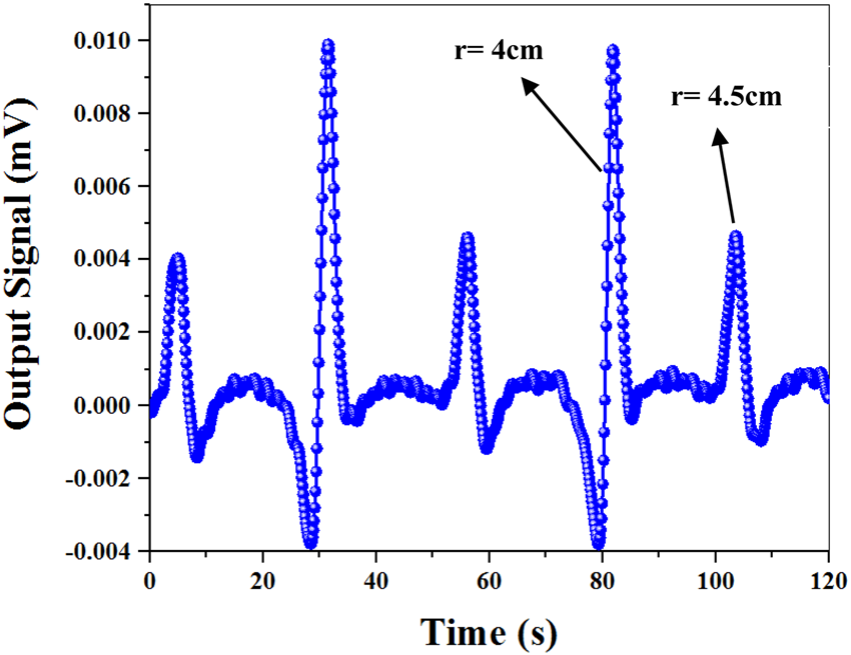
Experimental result of the light intensity changes created by passing the magnetized microwire adjacent to the atomic magnetometer.

The near-resonance linear dependence of the in-phase signal was used to measure the projection of the microwire magnetic field along the direction of the bias magnetic field B_0_. An electronic phase-locked loop (PLL) was applied to ensure that the frequency has tracked Larmor frequency of B_0_ (f_0_ in Fig. 3(a)). Each time the microwire passed the atomic magnetometer, a spike-like signal was detected, as displayed in Fig. 4.

According to Fig. 1(b), when the vertical position of the microwire relative to the cell was 4 cm, a stronger signal was detected in Fig. 4. Furthermore, the weaker signal was related to passage of the microwire with a 4.5 cm vertical position relative to the cell. It is obvious that the signal intensity has decreased upon reduction in the vertical distance, r.

### Analyzing the spike-like signal of the moving magnetic microwire

Here, we needed to know how the magnetic field and local gradient field of the microwire affected the atomic magnetometer output signal. For clarification, we analyzed the dependent parameters of the magnetic resonance output signal. The typical magnetic resonance signal, as shown in Fig. 3(a), fits the in-phase and quadrature Lorentzian line-shape^19,20,38,41^. The FWHM of the quadrature signal which is equal to the peak-to-peak frequency interval of the in-phase curve is given by:

2 $$\Gamma =\frac{1}{{{\rm{T}}}_{2}}\sqrt{{1+({\rm{\gamma }}{\rm{B}}}_{1}{)}^{2}{{\rm{T}}}_{1}{{\rm{T}}}_{2}}$$

In this relation, γ represents the gyromagnetic ratio of the atomic spin. B_1_ is the amplitude of excitation magnetic field which is equal to 10 nT. T_1_ and T_2_ denote the longitudinal and transverse relaxation times, respectively:

3 $$\frac{1}{{{\rm{T}}}_{1}}=\frac{1}{{\rm{q}}}({{\rm{R}}}_{{\rm{SD}}}+{{\rm{R}}}_{{\rm{OP}}})+{{\rm{R}}}_{{\rm{Wall}}}$$

4 $$\frac{1}{{{\rm{T}}}_{2}}=\frac{1}{{{\rm{T}}}_{1}}+\frac{1}{{{\rm{q}}}_{{\rm{SE}}}}{{\rm{R}}}_{{\rm{SE}}}+{{\rm{R}}}_{{\rm{gr}}}$$

In the longitudinal component of the atomic spin relaxation rate, q is the slowing-down factor in the nuclear spin. This factor is given by, q = 2I + 1 (at high polarization) and demonstrates how the atomic coherence is maintained after destroying electron spins. R_OP_ is the optical pumping rate which changes the atom’s angular momentum along the pumping direction. The second term, R_SD_, is the spin destruction rate which can be calculated by: ${{\rm{R}}}_{{\rm{SD}}}={{\rm{R}}}_{{\rm{SD}}}^{{\rm{self}}}+{{\rm{R}}}_{{\rm{SD}}}^{{\rm{B}}}+{{\rm{R}}}_{{\rm{SD}}}^{{\rm{Q}}}$, where these three terms indicate the rate of collisions with other alkali atoms, buffer gas atoms, and N_2_ gas molecules, respectively. The third term in the equation of T_1_, is a relaxation term due to the vapor cell wall collisions.

In the relation of the transverse relaxation time T_2_, the second term indicates the contribution of spin-exchange collisions, R_SE_. Such collisions are due to an interaction between attractive singlet and repulsive triplet components of the electron spins whose contribution to T_2_ is determined by the spin-exchange broadening factor, q_SE_. This factor is dependent on the magnetic field strength such that its inversion, 1/q_SE_, approaches zero when the magnetic field is zero^19,20,38^. The parameter R_gr_ in the T_2_ relation demonstrates the sensitivity of the relaxation rate to the magnetic field gradient that has been studied in buffer gas cells as well as buffer-gas-free antirelaxation-coated cells both theoretically and experimentally in previous studies^26,27^.

At this stage, the spike-like signal is analyzed while observing changes in the relaxation rate. We know that the longitudinal relaxation rate is invariant when the moving microwire passes the cell (as the optical pumping rate, the spin destruction rate, and the wall collision rate are fixed). According to the Γ relation, as the excitation magnetic field B_1_ and the longitudinal relaxation rate T_1_ were invariant in our experiments, we assumed that the changes of the light intensity were attributed to the T_2_ changes. As a result, the FWHM of the magnetic resonance curve was inversely proportional to the transverse relaxation time, Γα1/T_2_; thus, the slope of the in-phase Lorentzian line-shape was proportional to the transverse relaxation time, mαT_2_.

**Figure 5:**
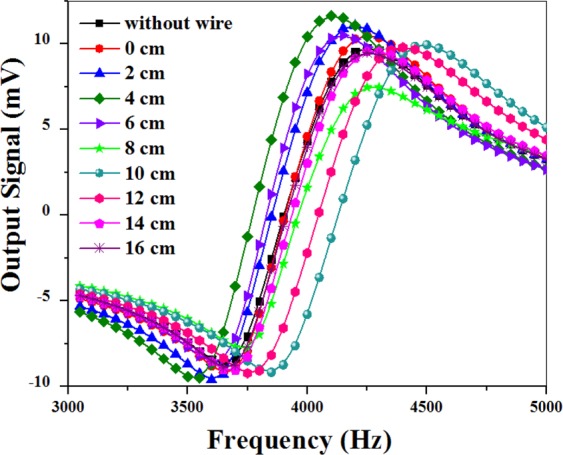
In-phase signal recorded in the absence of magnetic microwire (black squares) and in-phase signal recorded in the presence of magnetic microwire at 0 cm (red circles), 2 cm (blue triangles), 4 cm (olive squares), 6 cm (violet triangles), 8 cm (green stars), 10 cm (cyan circles), 12 cm (pink hexagons), 14 cm (magenta pentagonals), and 16 cm (purple stars) positions on the graded path.

**Figure 6:**
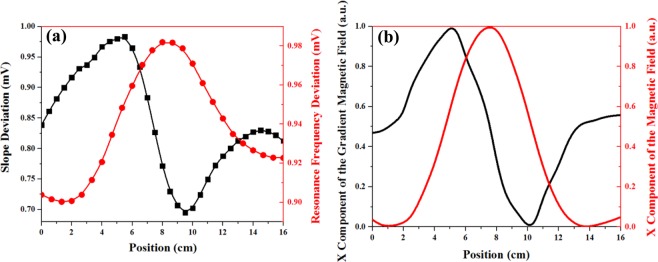
(**a**) Modification of the slope (black squares) and frequency of the signals (red circles) obtained in different positions of the microwire; (**b**) Simulated X component of the magnetic field (red solid line) and its inhomogeneous local magnetic field gradient (black solid line) versus different positions of the microwire.

To investigate this assumption thoroughly, the resonance curves associated with the presence of the microwire in the specified positions on the graded path are shown in Fig. 5. This figure indicates that the presence of the microwire affects some parameters such as the slope (m) and the turning point frequency (resonance frequency of spins (dF)) of the magnetic resonance curves. Variations of these parameters in different positions were extracted from Fig. 5 and depicted in Fig. 6(a).

It is clear that the presence of the microwire in different positions can divert the bias magnetic field in the linear region and ultimately changes the resonance frequency f_0_.

5 $${f}_{{\rm{prec}}}={\rm{\gamma }}({\overrightarrow{{\rm{B}}}}_{0}+{\rm{\delta }}{\overrightarrow{{\rm{B}}}}_{{\rm{Wire}}})={{\rm{f}}}_{0}{+{\rm{\delta }}{\rm{f}}}_{{\rm{wire}}}$$

In this relation, any deviation from the resonance frequency of f_0_ (δf_Wire_) is proportional to the magnetic field of the microwire, δB_Wire_. In other words, δB_Wire_ can deviate the turning point frequency of the curves (the red circles in Fig. 6(a))^17^. To obtain variations of the magnetic field of the microwire, we calculated B_x_ versus different positions along the *x* direction through the simulation (the red solid line in Fig. 6(b)). Note that the X component of the magnetic field (B_x_) was the dominant term, being 4 orders of magnitude larger than the other components. The trend we observed in the resonance frequency deviations is similar to B_x_.

In addition, the inhomogeneous local magnetic field gradient produced by the moving microwire can influence the slope of the magnetic resonance curve^23–27^. The black solid line in Fig. 6(b) represent the variations in the inhomogeneous local magnetic field gradient, dB_x_/dx in terms of the microwire’s positions obtained by the simulation. It is remarkable that the trend of the slope deviations (Fig. 6(a)) is similar to the curves of the local gradient field of the moving magnetic microwire.

**Figure 7:**
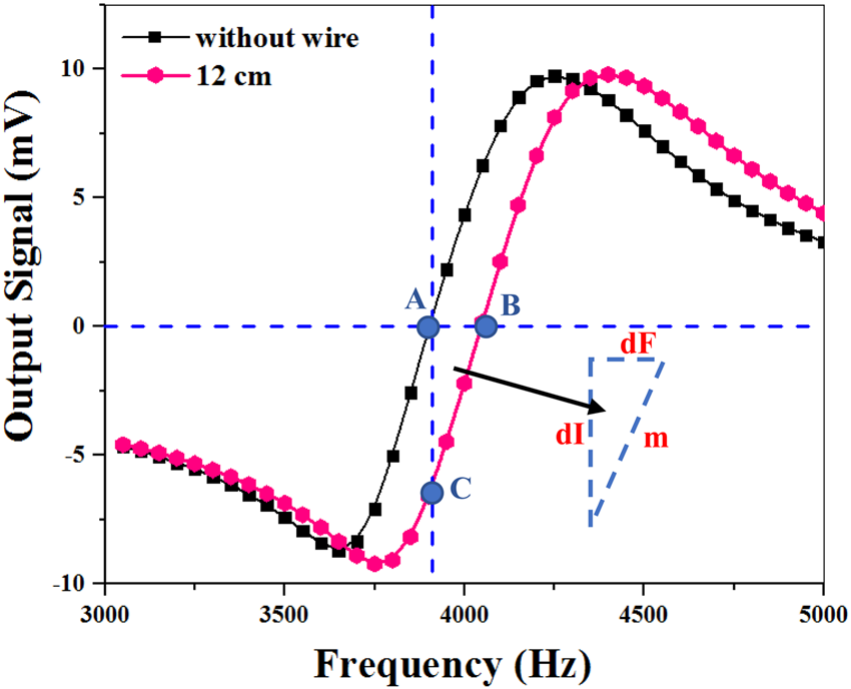
Modification of the slope (black squares) and frequency of the signals (pink hexagons) obtained in different positions of the microwire.

The simultaneous changes of m and dF in different positions can alter the light intensity. For more detailed study of the spike-like signal, we selected one of the in-phase signals (in Fig. 5) related to the presence of the microwire 12 cm away from the origin, x = 0 (the pink hexagons in Fig. 7). Then, this curve was compared with the curve obtained in the absence of the microwire (the black squares in Fig. 7). The right triangle, ABC in Fig. 7, is considered as an indicator of the slope and turning point frequency deviations of the curve. In this triangle, dI denotes the modification of the light intensity in the linear region resulting from the multiplication of the slope (m) by the detuning from the resonance frequency (dF=f-f_0_) in one single position.

**Figure 8:**
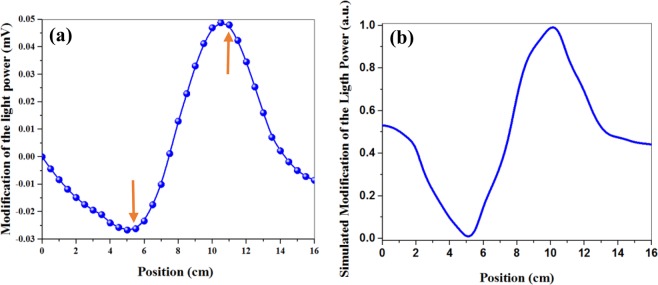
(**a**) Experimental results of the light intensity changes obtained through multiplying the slope curve by the resonance frequency curve versus different positions. The arrow marks represent the sensor location located between 5.5 and 10.5 cm; (**b**) Multiplication of the magnetic curve by the gradient field curve versus different positions of the microwire.

To obtain dI for all other microwire positions, we calculated changes in the slope (m) and turning point frequency deviation of the curves (dF) for different positions of the microwire in Fig. 6(a). Through multiplying m by dF, we can obtain dI for all positions. The final result (as shown in Fig. 8(a)) is found to be similar to the spike-like signal in Fig. 4. The minimum, maximum, and turning points of the curve almost correspond to the microwire arrival to the first edge, second edge, and center of the cylindrical alkali vapor cell, respectively (Fig. 1(b)). In addition, the simulation values of B_x_ have been multiplied by dB_x_/dx for comparison with experimental results. Figure 8(b) indicates a behavior similar to the spike-like signal in Fig. 4. Accordingly, the numerical simulations and experimental results are in a good qualitative agreement.

### Dependency of the signal on the length of the magnetic microwire

**Figure 9:**
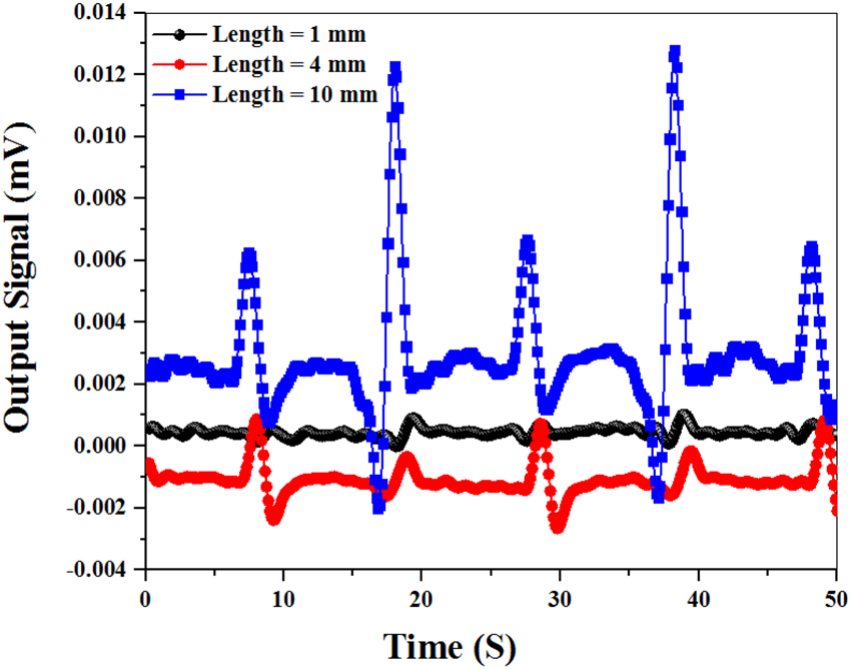
Experimental results of the changes in the light intensity for three lengths of the microwire.

In the previous section, we studied the factors generating the spike-like signal. Now one of the main problems is to estimate the limit of the microwire length which could be detected by the atomic magnetometer. To this end, three magnetic microwires with different lengths were chosen. The spike-like signals were recorded each time the magnetized microwires passing the atomic magnetometer at a constant velocity of 1.37 cm/s. The changes in the output light intensity over time are shown in Fig. 9. It can be seen that the light intensity diminishes by reducing the length of the microwire as the magnetic field of the microwire, δB_Wire_, declines. It is noticeable that all three curves exhibit the same peak to peak time intervals that are not dependent on the length of the microwire and its magnetic field. The smallest size of the magnetic microwire detected by the atomic magnetometer is equal to 1 mm.

### Dependency of the signal on the velocity

**Figure 10:**
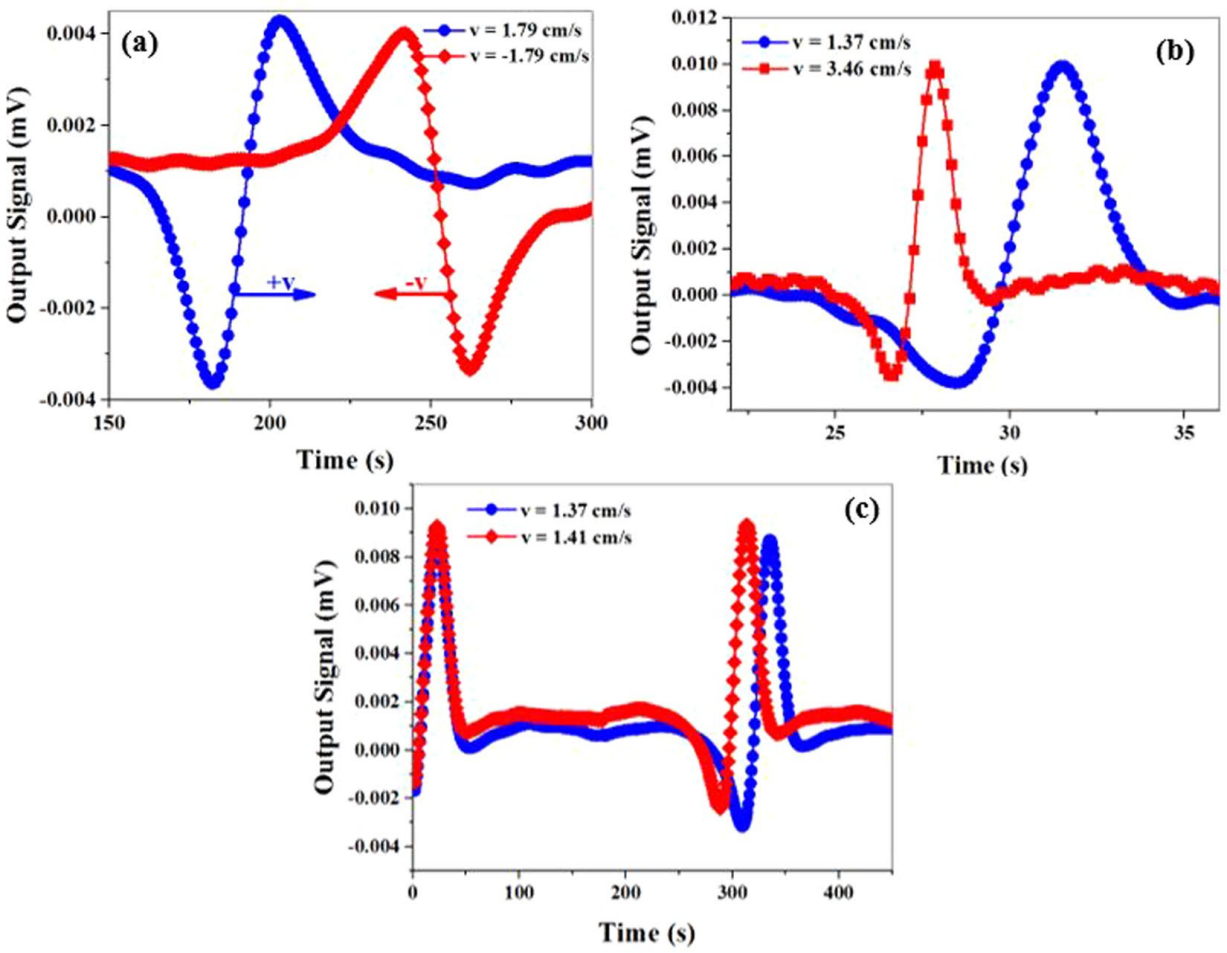
(**a**) Experimental results of the changes in the light intensity for two directions of the microwire motion; (**b**) Changes in the transmitted light for two velocities of 1.37 cm/s and 3.46 cm/s; (**c**) The accuracy of the velocimetry.

Now, we intend to show the dependency of the signal on the velocity direction, which will be then proved to be the main advantage of measuring the inhomogeneous local magnetic field gradient. To this aim, we detected the changes in the transmitted light for two opposite velocities, +1.79 cm/s and −1.79 cm/s. As illustrated in Fig. 10(a), the amplitude of the curve has been inverted upon alteration in the velocity direction. This output signal in Fig. 10(a) (blue circles) has resulted from simultaneous changes in the magnetic field and inhomogeneous local magnetic field gradient of the microwire in different positions (as shown in Fig. 8(a)). When the microwire moves along the bias magnetic field and passes the first edge of the vapor cell, a minimum is observed. Next, when the microwire reaches the second edge, the maximum of the curve is observed. However, when the motion direction is opposite to the bias magnetic field (return path), an inverse behavior is obtained. As the magnetic field and inhomogeneous local magnetic field gradient of the microwire are the same as before, in the beginning of the return path, the microwire passes the second edge of the vapor cell while the maximum of the curve is observed. Then, the microwire reaches the first edge of the vapor cell and produces a minimum behavior in the curve, as shown in Fig. 10(a) (red squares).

To understand the effect of the velocity amplitude on the output signal, the changes in the transmitted light were detected for two velocities, 1.37 cm/s and 3.46 cm/s. According to Fig. 10(b), there is no significant direct relationship between the amplitude of the velocity and intensity of the output signal. On the other hand, the peak-to-peak time intervals of the curves are 3 s and 1.2 s which are in accordance with 4.11 cm and 4.15 cm distances travelled in space. The result suggest that the peak-to-peak space intervals are in the same order and do not depend on the velocity changes.

One of the practical issues in tracking a magnetic object is to identify the accuracy of the velocimetry. To investigate this, the changes in the transmitted light intensity were detected for the microwire at a velocity of 1.41 cm/s. Then, the velocity was reduced to the extent that a separable output signal was obtained for a velocity of 1.37 cm/s (Fig. 10(c)). The results reveal that the accuracy of the velocimetry is about 40 µm/s.

### Dependency of the signal on the vapor cell size

**Figure 11:**
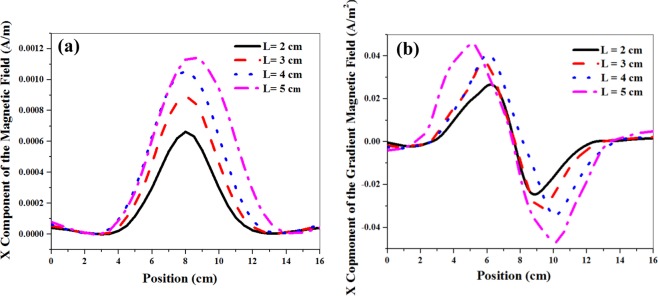
(**a**) Simulated X component of the magnetic field; and (**b**) Simulated X component of the gradient magnetic field for different positions of the magnetic microwire for four different lengths of the cell.

In the present section, we study the advantages and limitations of the large alkali vapor cell size for the magnetic detection. We intend to investigate the dependency of the atomic magnetometer signal on the size of the vapor cell via the simulation calculations. As mentioned previously, the circularly polarized light transmitted through the cell probes an average of the magnetic field throughout. So, we have assumed in the simulation that the averaging length is proportional to the size of the vapor cell. The averaged magnetic field and inhomogeneous local gradients of the magnetic microwires are shown in Fig. 11(a,b) for different microwire positions and four various lengths of the cell. The figure shows that the amplitude of the magnetic field and its local gradient grow when the averaging length (the length of the cell) increases. Furthermore, the peak-to-peak space interval of the local gradient curves becomes more distinguishable. As this interval is only dependent on the length of the cell, we can detect the velocity direction more effectively by enlarging the cell size.

**Figure 12:**
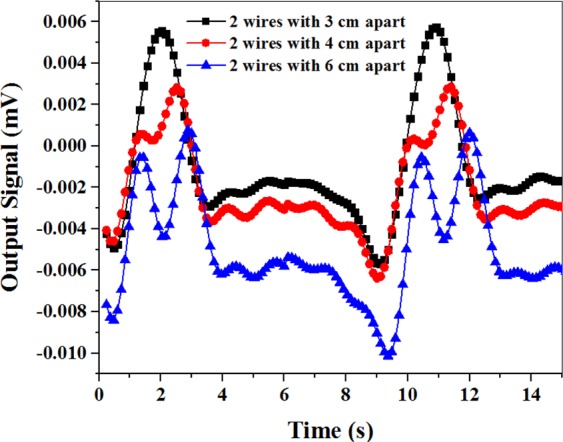
Measurement of the spatial resolution of the atomic magnetometer.

Although, increasing the cell size improves the output signal and provides detection of the velocity direction, but deteriorates the spatial resolution. So, we need an optimum size of the vapor cell. To investigate the effect of the large cell on the spatial resolution, two magnetic microwires were loaded on the belt. Figure 12 shows that two distinct peaks are generated in the output signal when the microwires are 6 cm apart. By reducing the distance between the microwires, two peaks were merged into a single peak. The figure suggests that the distinction of the two wires is possible when the distance between them is more than 4 cm.

## Conclusion

Atomic magnetometers open new ways to designing a high-sensitivity portable magnetometer which is useful in various applications such as magnetic object tracking. Lack of sufficient information about the dependence of the atomic magnetometer signal on the tracking parameters of the object is one of the issues that has remained neglected so far. In this paper, we detected a moving magnetic object which was placed in the static magnetic field using an Mx-atomic magnetometer. The experimental results showed that the simultaneous changes in the magnetic field and its inhomogeneous local gradient due to the moving magnetic microwire alter the light intensity causing a spike-like signal. This signal was obtained by studying the changes in the slope (m) and turning point frequency deviation of the resonance curves associated with the presence of the magnetic microwire in the specified positions of the transfer path. The magnetic field and local gradient field of the microwire were calculated through finite element numerical simulations which confirmed the experimental results.

Dependency of the output signal on the velocity parameters was investigated in detail, too. Notably, although this atomic magnetometer is scalar in nature, it was sensitive to the direction of the velocity. The accuracy of the velocimetry was obtained as 40 µm/s which may be practical in some biomedical techniques. Furthermore, the intensity and peak-to-peak space interval of the signal did not depend on the velocity amplitude, but they were associated with the alkali vapor cell size. Thus, by considering the spatial resolution, we need an optimum size of the vapor cell for accurate magnetic object tracking.
